# Bilirubin Alleviates Spinal Cord Injury by Enhancing SOCS3‐Mediated Anti‐Inflammatory Effects via Gas6‐Axl Signaling

**DOI:** 10.1111/cns.70538

**Published:** 2025-09-17

**Authors:** Kun‐Mao Jiang, Ya‐Qi Luan, Na Shen, Xiu‐Ting Qi, Liang Hu, Yu Wang, Wen‐Tao Liu, Rong Wang, Tong‐Tong Lin, Da‐Yong Peng

**Affiliations:** ^1^ Department of Nephrology Shandong Provincial Hospital Affiliated to Shandong First Medical University Jinan Shandong China; ^2^ Jiangsu Key Laboratory of Neurodegeneration, Department of Pharmacology Nanjing Medical University Nanjing Jiangsu China; ^3^ Rehabilitation Medicine Center The First Afliated Hospital of Nanjing Medical University Nanjing Jiangsu China; ^4^ State Key Laboratory of Technologies for Chinese Medicine Pharmaceutical Process Control and Intelligent Manufacture Nanjing University of Chinese Medicine Nanjing Jiangsu China; ^5^ Department of Orthopedic Surgery The First Affiliated Hospital of Shandong First Medical University & Shangdong Provincial Qianfoshan Hospital Jinan Shandong China

**Keywords:** Axl, bilirubin, Gas6, microglia, neuroinflammation, spinal cord injury

## Abstract

**Background:**

Many studies have emphasized the role of microglia‐mediated neuroinflammation in spinal cord injury (SCI); however, effective clinical targets remain elusive. The growth arrest‐specific 6 (Gas6)/Axl receptor tyrosine kinase (Axl) signaling pathway has been implicated in reducing inflammation, promoting tissue repair, and functional recovery. Here, we elucidate the importance of the Gas6‐Axl signaling pathway in SCI repair and evaluate the role of bilirubin in modulating Gas6‐Axl signaling after SCI.

**Methods:**

SCI mice model was used to investigate the effects of bilirubin treatment on inflammation and motor function recovery. Additionally, Gas6‐deficient (*Gas6* ^*−*^ */* ^*−*^) mice and wild‐type (WT) mice were employed to examine the role of Gas6‐Axl signaling in SCI recovery. Microglial cells were cultured to assess the effects of bilirubin on the activation of the Gas6‐Axl‐SOCS3 signaling pathway.

**Results:**

*Gas6* ^*−*^ */* ^*−*^ mice exhibited increased mortality, severe locomotor deficits, and impaired neuromuscular activity compared to WT mice. Bilirubin treatment in SCI models facilitated recovery by upregulating Gas6‐Axl signaling, which in turn enhanced SOCS3 expression and suppressed the expression of pro‐inflammatory mediators such as IL‐1β and MMP‐9. Furthermore, bilirubin treatment reduced microglial activation, highlighting its neuroprotective and anti‐inflammatory properties.

**Conclusions:**

This study underscores the importance of the Gas6‐Axl‐SOCS3 axis in regulating functional recovery and inflammation after SCI. Activation of the Gas6‐Axl pathway, particularly when combined with bilirubin treatment, represents a promising therapeutic strategy for mitigating SCI‐induced damage and improving functional outcomes. Given their central role in both the pathogenesis and resolution of SCI, bilirubin treatment emerges as a promising clinical therapeutic drug for SCI.

## Graphical abstract

Schematic illustration depicting bilirubin activation of the Gas6/Axl/SOCS3 pathway to reduce neuroinflammation and enhance spinal cord recovery.

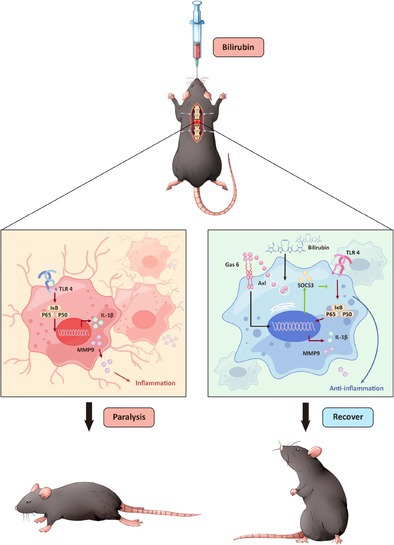

## 1 Introduction

Spinal cord injury (SCI) remains a devastating neurological condition, often causing permanent impairment and significantly reducing patients' quality of life [1, 2]. Current treatment primarily relies on surgical interventions, including spinal realignment, stabilization, and decompression of neural structures, which aim to prevent secondary damage but have limited capacity to restore lost neurological function [3]. In the acute phase, methylprednisolone is sometimes administered to mitigate inflammation, though its clinical benefits remain controversial due to inconsistent efficacy and potential adverse effects [4]. These limitations highlight a critical unmet need in SCI management: while existing approaches address structural stabilization and short‐term neuroprotection, they fail to target the underlying pathological cascades—including chronic neuroinflammation, oxidative stress, and progressive neurodegeneration—that drive long‐term disability. Consequently, there is an urgent demand for novel pharmacological strategies that can modulate these fundamental mechanisms and promote meaningful neurological recovery.

SCI leads to profound neurological deficits due to both primary mechanical injury and secondary injury processes [4]. Among the major contributors to secondary injury is microglia‐mediated neuroinflammation, which plays a pivotal role in tissue damage progression and impedes recovery [5, 6, 7, 8]. Initially, microglial activation serves a protective role, facilitating the clearance of cellular debris and preventing additional injury. However, prolonged or excessive activation can shift this response to chronic inflammation, exacerbating neuronal damage and hindering tissue repair [5, 9, 10]. As a result, strategies aimed at inhibiting microglial activation and mitigating subsequent inflammatory responses are considered crucial for enhancing SCI recovery.

The Growth arrest‐specific 6‐Axl receptor tyrosine kinase (Gas6‐Axl)signaling pathway has emerged as a critical regulator of inflammation, cell survival, and tissue repair in various injury models [11, 12]. Recent studies have highlighted its role in reducing neuroinflammation and promoting tissue regeneration after injury, making it a promising target for SCI therapy [13]. Given its involvement in both inflammation modulation and neuroprotection, the Gas6‐Axl pathway represents a compelling therapeutic target for SCI, and strategies aimed at enhancing its signaling could offer a novel approach to reduce neuroinflammation and promote functional recovery following SCI.

Bilirubin, a degradation product of hemoglobin, has historically been viewed primarily as a harmful metabolic byproduct [14]. However, recent evidence has underscored its potent antioxidant, anti‐inflammatory, and neuroprotective properties [15, 16, 17]. As an endogenous antioxidant, bilirubin scavenges free radicals, thereby reducing oxidative stress‐induced neuronal damage. Furthermore, bilirubin has been shown to suppress inflammatory responses by inhibiting the release of pro‐inflammatory cytokines, such as Tumor necrosis factor‐α (TNF‐α), Interleukin‐1β (IL‐1β), and Interleukin‐6 (IL‐6), thereby preserving neuronal integrity [18]. Despite these promising effects, the precise molecular mechanisms underlying bilirubin's anti‐inflammatory actions remain unclear.

Given the lack of effective pharmacological interventions for SCI and the potential of bilirubin to modulate inflammation and oxidative stress, this study aims to explore whether bilirubin can enhance its anti‐inflammatory effects through the Gas6‐Axl signaling pathway, ultimately improving SCI recovery outcomes. By investigating bilirubin's potential role in enhancing Gas6‐Axl signaling, this study seeks to fill a critical gap in SCI therapeutics, providing a foundation for future pharmacological strategies targeting inflammation and neuroprotection.

## 2 Materials and Methods

### 2.1 Animals and Treatment

Eight‐week‐old female C57BL/6J mice (18–22 g) were purchased from the Experimental Animal Center at Nanjing Medical University, China. Mice were kept under standard housing conditions, including a controlled environment of 22°C ± 2°C, a 12‐h light/dark cycle, and 55% ± 5% relative humidity. Experimental protocols were approved by the Institutional Animal Care and Use Committee (IACUC‐2211021) and conducted following ethical guidelines for animal research.

### 2.2 SCI Induction and Bilirubin Treatment

SCI was induced using an established contusion model as previously described [19]. Adult female mice were anesthetized via intraperitoneal injection of 1% (w/v) pentobarbital sodium (50 mg/kg), and a T9 laminectomy was performed to expose the spinal cord. The injury was produced using the Allen's impactor device (RWD, China), which delivered a controlled force of 60 kilodynes to ensure consistent severity. In the sham‐operated control group, only the laminectomy procedure was performed. After the injury, bilirubin was administered via oral gavage at a dose of 30 mg/kg every other day over a 5‐week period, starting immediately post‐injury. All animals were closely monitored for signs of distress and recovery throughout the study. The injury severity was standardized by controlling the force and depth of the impactor, and the experiment ensured consistent injury induction across all groups.

### 2.3 Behavioral Test

Motor function recovery was evaluated using the Basso Mouse Scale (BMS) test and gait analysis. The BMS open field test and automated gait analysis followed standardized protocols from previous SCI studies [20, 21]. Behavioral tests were conducted at 7, 14, 21, 28, and 35 days post‐SCI by two blinded experimenters. The BMS test was conducted in a 1 m × 1 m open field, and all mice were acclimated to the testing environment before the formal assessment. The BMS scoring system ranged from 0 to 9, with higher scores indicating better motor function recovery in SCI mice. Hind limb motor function was assessed at 35 dpi using the CatWalk gait analysis system. All mice were performed to record at least three consecutive walking cycles. To prevent interference from external light during the gait analysis, the experiment was conducted in a darkened environment to ensure clear footprint recordings. Gait parameters were automatically analyzed using the WalkAnalysator software, followed by a manual, frame‐by‐frame inspection and correction to guarantee result accuracy.

### 2.4 H‐Reflex and EMG

An electromyographic evoked potential system (Hai Shen, Shanghai, China) was used to evaluate the H‐reflex and electromyographic (EMG) activity 35 days after SCI surgery. For H‐reflex assessment, a stimulating electrode was inserted at the sciatic nerve exit, and a recording electrode was placed in the gastrocnemius muscle. For EMG recording, the stimulating electrode was inserted into the skin over the muscle, and the recording electrode was inserted into the belly of the gastrocnemius muscle. The reference electrode was placed on the muscle tendon or nearby non‐muscle area, and the ground electrode was inserted into the tail. EMG activity was recorded during voluntary movement and passive stimulation, and the signals were processed for analysis.

### 2.5 Western Blotting

Spinal cord tissues (T9‐T11) were homogenized in RIPA lysis buffer, and the lysates were centrifuged at 12,000 g for 15 min at 4°C to collect supernatants. Protein concentrations were measured using the BCA assay. The proteins were separated by 8%–12% SDS‐PAGE and transferred onto PVDF membranes (Millipore, USA). Membranes were blocked with 5% milk for 2 h at room temperature, followed by incubation with primary antibodies overnight at 4°C. The primary antibodies included Gas6 (1:1000, A8545, ABclonal), p‐Axl (1:1000, 44463s, Cell Signaling Technology), Axl (1:1000, A22378; ABclonal), SOCS3 (1:1000, A21981; ABclonal), MMP‐9 (1:1000, 10375‐2‐AP; Proteintech), and IL‐1β (1:1000, I3767; Sigma‐Aldrich). After TBST washing, membranes were treated with horseradish peroxidase‐conjugated secondary antibodies for 2 h at room temperature. Protein bands were detected using enhanced chemiluminescence, and their intensity was quantified with Image J software.

### 2.6 Cell Culture and Treatment

BV‐2 microglial cells were cultured in Dulbecco's modified Eagle's medium supplemented with 10% (v/v) fetal bovine serum, 100 U/mL penicillin, and 100 U/mL streptomycin (KeyGEN, China). Cells were maintained in a humidified atmosphere containing 5% CO_2_ at 37°C. For treatment, the cells were incubated with various concentrations of bilirubin (0.1, 1, and 10 μM) for 12 h to assess dose‐dependent effects on Gas6 and p‐Axl expression. To evaluate the effect of Axl inhibition on bilirubin‐induced SOCS3 expression, the Axl inhibitor R428 (Selleckchem, S7286) was added to the culture at a concentration of 1 μM 1 h prior to bilirubin treatment. After treatment, cells were harvested for further analysis.

### 2.7 Immunofluorescence and Imaging

The T8‐T10 spinal cord tissues were collected and then further fixed in 4% paraformaldehyde for 24 h. The spinal tissues were embedded in paraffin and cut into 5‐μm sections. After deparaffinization and rehydration, the sections were performed by immunohistochemistry. Briefly, the sections were blocked for 1 h by PBS with 10% donkey serum albumin and 0.3% Triton X‐100 at room temperature, and then the sections were washed with PBS and incubated overnight with primary antibodies at 4°C. The following primary antibody, Iba1, was used. Subsequently, the sections were incubated with secondary antibodies for 2 h at room temperature. Finally, the sections were photographed by a Zeiss LSM800 confocal microscope, and the image was acquired and processed with Zeiss.

### 2.8 Statistical Analysis

GraphPad Prism version 9.0 (GraphPad Software Inc., USA) was used for statistical analysis. The experimental data were shown as the mean ± SEMs. No data were excluded from the data analysis. The normal distribution of the data was determined by the Shapiro–Wilk normality test. For statistical comparisons, Student's *t* test (between two groups), one‐way ANOVA (between multiple groups) with Tukey's test (equal variances), or two‐way ANOVA with Tukey's test (between multiple variables) was used to compare normally distributed variables. *p* < 0.05 was considered significant.

## 3 Results

### 3.1 Bilirubin Treatment Improves Motor Function After SCI in Mice

**FIGURE 1:**
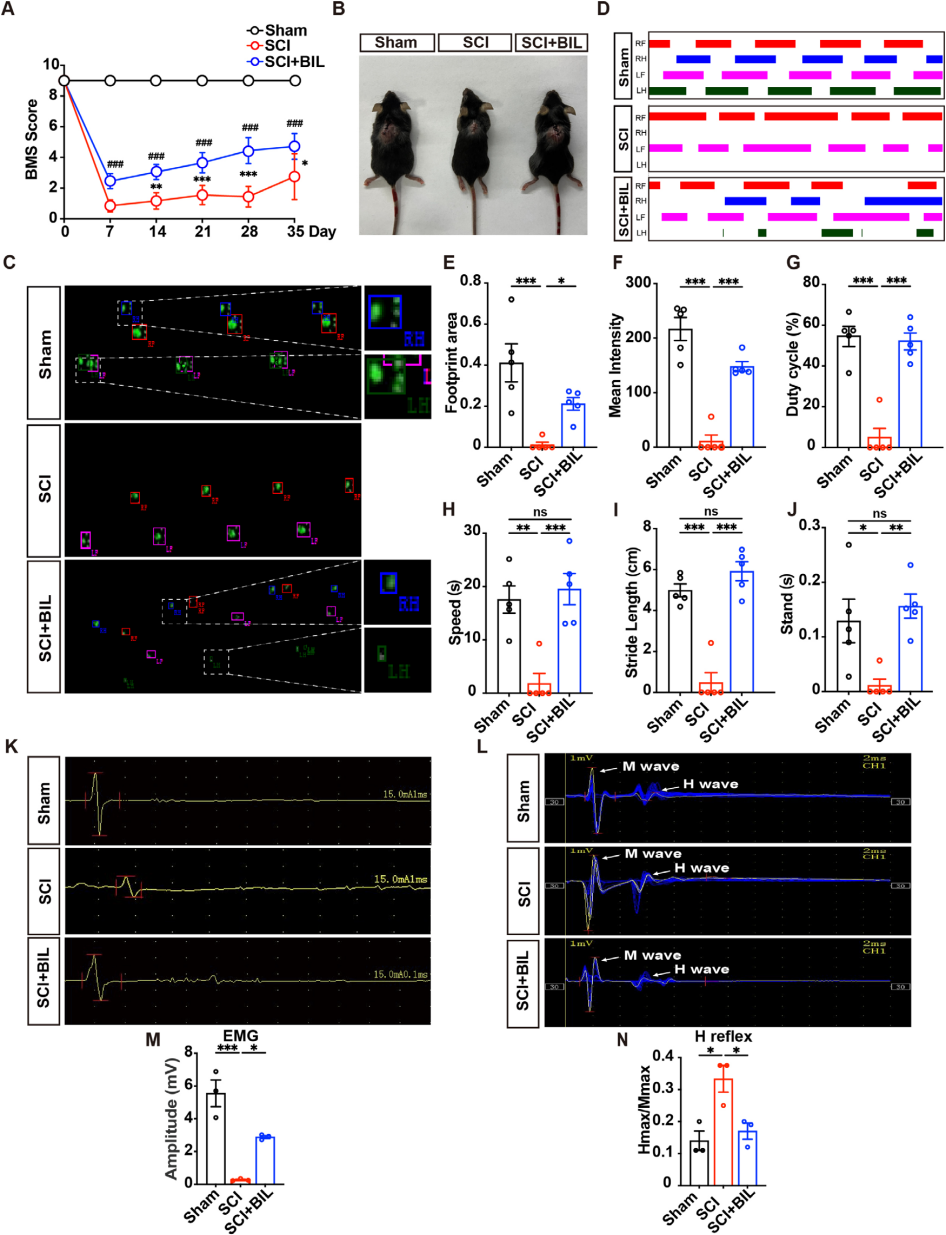
Bilirubin treatment improves motor function after spinal cord injury in mice. (A) Basso Mouse Scale (BMS) scores were used to assess locomotor function over 5 weeks post‐SCI (*n* = 5, the *p* values were determined by Two‐way ANOVA with Tukey's multiple comparisons test, *p < 0.05, **p < 0.01, ***p < 0.001 vs. sham group; ###*p* < 0.001 vs. SCI group). (B) Representative images show the flexion state of the hind limbs in mice from the sham, SCI, and SCI + BIL groups at 35 days after SCI. (C) Representative footprint intensity images for each group were collected at 35 days post‐SCI (*n* = 5). (D) Representative timing view plots from the CatWalk system show the temporal pattern of paw contact with the ground for each limb across different groups. The length of each colored bar represents the duration of ground contact during the gait cycle. LF, left front; LH, left hind; RF, right front; RH, right hind. (E–J) Quantitative analysis of gait parameters using the CatWalk system in each group. The SCI + BIL group exhibited significant improvements in gait parameters at 35 days post‐SCI. The following gait parameters were quantitatively analyzed: (E) footprint area, (F) mean intensity, (G) duty cycle, (H) speed, (I) stride length, and (J) stand duration (*n* = 3). (K) Representative electromyography (EMG) images were obtained 35 days post‐SCI (*n* = 3). (L) Representative H‐reflex recordings showing M wave and H wave images were obtained 35 days post‐SCI (*n* = 3). (M, N) EMG amplitude and the H‐max/M‐max ratio were quantified for each group (*n* = 3). All data are presented as the means ± SEMs. In (E–J), (M), and (N) the *p* values were determined by one‐way ANOVA with Tukey's post hoc test, *p < 0.05, **p < 0.01, ***p < 0.001.

Firstly, we investigated the effect of bilirubin on a SCI mice model. The BMS was used to evaluate hindlimb motor function recovery over a 5‐week period post‐SCI. Bilirubin treatment led to a significant enhancement in motor function, observable as early as the second week post‐injury compared to the SCI group. This improvement persisted and progressively increased throughout the 5‐week observation period (Figure 1A). In addition, the flexion state of the hind limbs in mice was significantly improved after bilirubin treatment (Figure 1B).

To further assess locomotor recovery, gait analysis was conducted using the CatWalk system (Figure 1C). Representative footprint intensity images and gait patterns demonstrated that SCI mice exhibited disrupted walking behavior, characterized by smaller and inconsistent footprint areas compared to the sham group. In contrast, mice treated with bilirubin showed markedly improved gait patterns, with larger and more stable footprints, indicating better recovery of hindlimb motor function. In addition, timing view plots (Figure 1D), which reflect the temporal sequence and duration of ground contact by each limb during the gait cycle, further illustrated the improvement in gait coordination after bilirubin treatment. SCI mice displayed irregular and shortened stance phases, whereas bilirubin‐treated mice exhibited more synchronized and prolonged contact times among all four limbs (LF, RF, LH, RH), indicative of enhanced gait stability and coordination. What is more, gait parameter analysis revealed that bilirubin treatment significantly improved parameters compared to the SCI group, including footprint area (E), mean intensity (F), duty cycle (G), speed (H), stride length (I) and stand duration (J).

In addition, bilirubin treatment significantly increased electromyography (EMG) amplitude in the SCI + BIL group, suggesting improved neuromuscular function (Figure 1K,L). Similarly, H‐reflex measurements (Figure 1M,N) showed that the SCI group had a reduced amplitude and Hmax/Mmax ratio, both of which were significantly improved by bilirubin treatment. This indicates partial restoration of reflex pathways.

### 3.2 Bilirubin Alleviates SCI by Increasing the Expression of SOCS3

**FIGURE 2:**
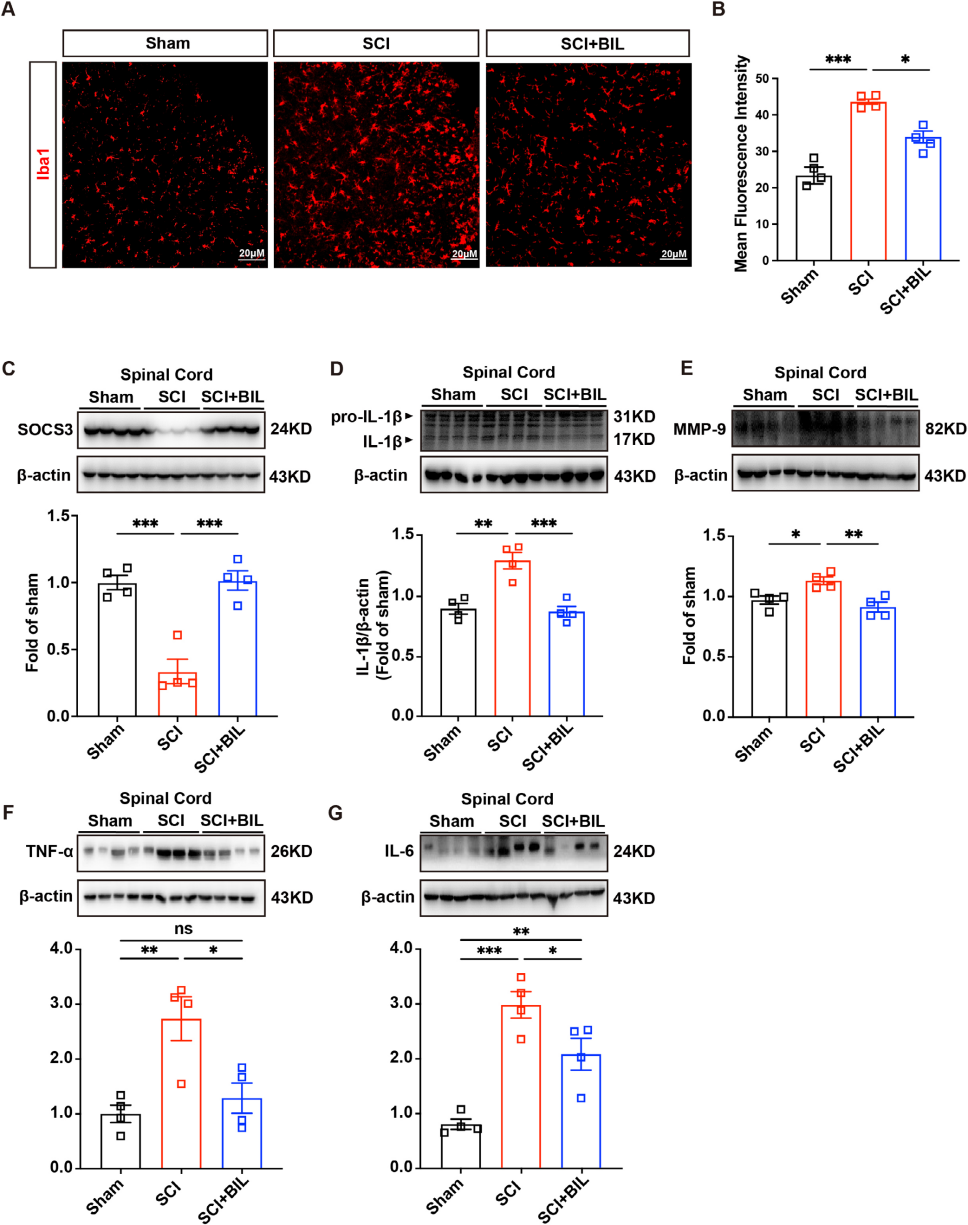
Bilirubin alleviates spinal cord injury by increasing the expression of SOCS3. (A, B) Immunofluorescence microscopy revealed Iba1 staining (red) in spinal cord sections 35 days post‐SCI. The quantification of the mean fluorescence intensity of Iba1 staining across the three experimental groups is presented (*n* = 4). Scale bars = 20 μm. (C–G) The levels of SOCS3, IL‐1β, IL‐6, TNF‐α and MMP‐9 proteins in the spinal cord were assessed by western blot 35 days after SCI (*n* = 4). All data are presented as the means ± SEMs. In (B–G), the *p* values were determined by one‐way ANOVA with Tukey's post hoc test, *p < 0.05, **p < 0.01, ***p < 0.001.

Microglia‐driven neuroinflammation is pivotal in SCI pathophysiology, contributing to secondary damage and worsening neurological dysfunction [22, 23]. After SCI, activated microglia release pro‐inflammatory factors, including tumor necrosis factor‐alpha (TNF‐α), interleukin‐1 beta (IL‐1β), and cyclooxygenase‐2 (COX‐2). These mediators amplify local inflammation and promote neuronal cell death [24]. Furthermore, activated microglia disturb the balance of matrix metalloproteinases (MMPs) in the spinal cord, leading to the degradation of the extracellular matrix and the breakdown of the blood‐spinal cord barrier (BSCB), which contributes to tissue damage and edema [25, 26]. In our study, we observed pronounced microglial activation and accumulation within 200 μm of the injury epicenter in SCI animals, as demonstrated by a significant increase in Iba1‐positive cells compared to sham controls. These activated microglia exhibited characteristic morphological alterations, including cell body hypertrophy and process retraction. Importantly, bilirubin treatment markedly reduced the density of Iba1‐positive microglia (Figure 2A,B). Additionally, bilirubin treatment decreased the expression of IL‐1β, IL‐6, TNF‐α and MMP‐9, indicating the suppression of inflammatory signaling pathways (Figure 2D–G).

We investigated the molecular mechanisms of bilirubin's effects by focusing on SOCS3, a crucial regulator of cytokine signaling and inflammation [27]. SOCS3 modulates immune responses through the suppression of pro‐inflammatory signaling pathways [28]. Our study showed that bilirubin treatment markedly increased SOCS3 expression in injured spinal cords, indicating that its anti‐inflammatory effects are partly mediated via SOCS3 upregulation (Figure 2C). These results underscore bilirubin's potential as a therapeutic agent to reduce neuroinflammation and enhance recovery after SCI, likely through SOCS3 signaling and microglial activation modulation.

### 3.3 Bilirubin Induces the Expression of SOCS3 via Activating the Gas6/Axl Signaling Pathway In Vitro

**FIGURE 3:**
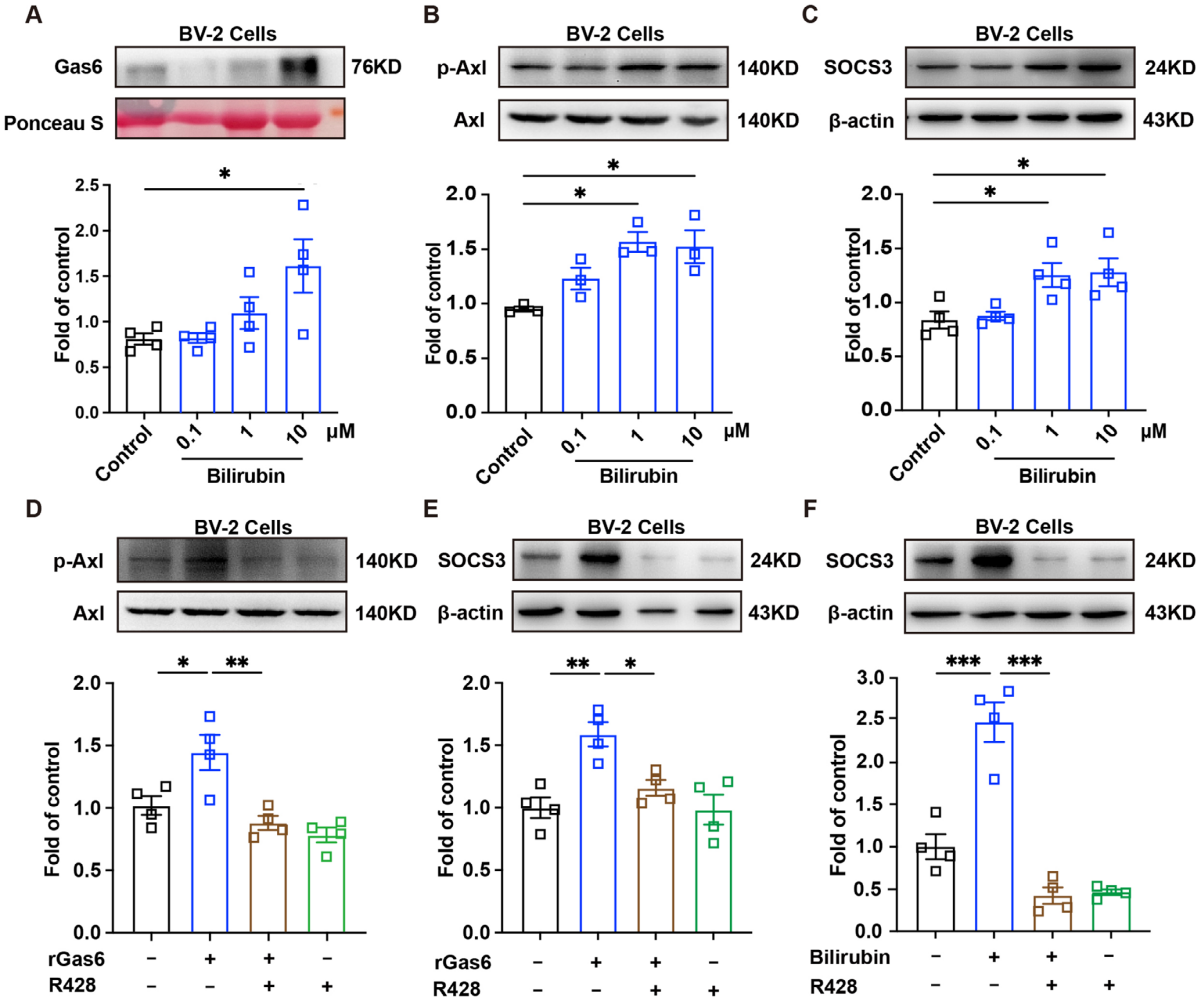
Bilirubin induces the expression of SOCS3 via activating the Gas6/Axl signaling pathway in vitro. (A) Supernatants from BV‐2 cells were collected 12 h post bilirubin treatment (0.1, 1, or 10 μM) for western blot analysis (*n* = 4). (B, C) Western blot results revealed the expression of p‐Axl and SOCS3 in BV‐2 cells treated with bilirubin (0.1, 1, or 10 μM) for 12 h (*n* = 4). (D, E) BV‐2 cells were pretreated with Axl inhibitor (R428, 1 μM) for 30 min, followed by coculture with recombinant Gas6 protein (rGas6, 500 ng/mL) for 6 h. The expression of p‐Axl and SOCS3 was analyzed for western blotting (*n* = 4). (F) Western blot demonstrated that Axl inhibitor (R428, 1 μM) suppressed bilirubin‐induced SOCS3 upregulation in BV‐2 cells (*n* = 4). All data are presented as the means ± SEMs. In (A–F), the *p* values were determined by one‐way ANOVA with Tukey's post hoc test, *p < 0.05, **p < 0.01, ***p < 0.001.

Previous studies have demonstrated that the Axl receptor can be activated in response to TAM ligands such as Gas6, which subsequently induces SOCS3 protein expression [29, 30]. To investigate this, Gas6 levels in the supernatant of BV‐2 cells treated with bilirubin at various concentrations (0.1, 1, and 10 μM) were measured. Results showed a significant increase in Gas6 expression 12 h after bilirubin treatment in BV‐2 cells (Figure 3A). Additionally, bilirubin promoted Axl receptor activation and upregulated SOCS3 expression under in vitro conditions (Figure 3B,C).

Furthermore, our findings revealed that treatment with exogenous recombinant Gas6 protein (rGas6, 500 ng/mL) for 2 h significantly activated the Axl receptor and enhanced SOCS3 protein expression in BV‐2 cells (Figure 3D,E). These effects, however, were abolished when cells were pretreated with the Axl inhibitor R428 (1 μM), indicating that bilirubin‐induced SOCS3 expression is dependent on Axl receptor activation (Figure 3E,F).

### 3.4 Ablation of the Gas6 Gene Eliminates the Therapeutic Effects of Bilirubin on SCI

**FIGURE 4:**
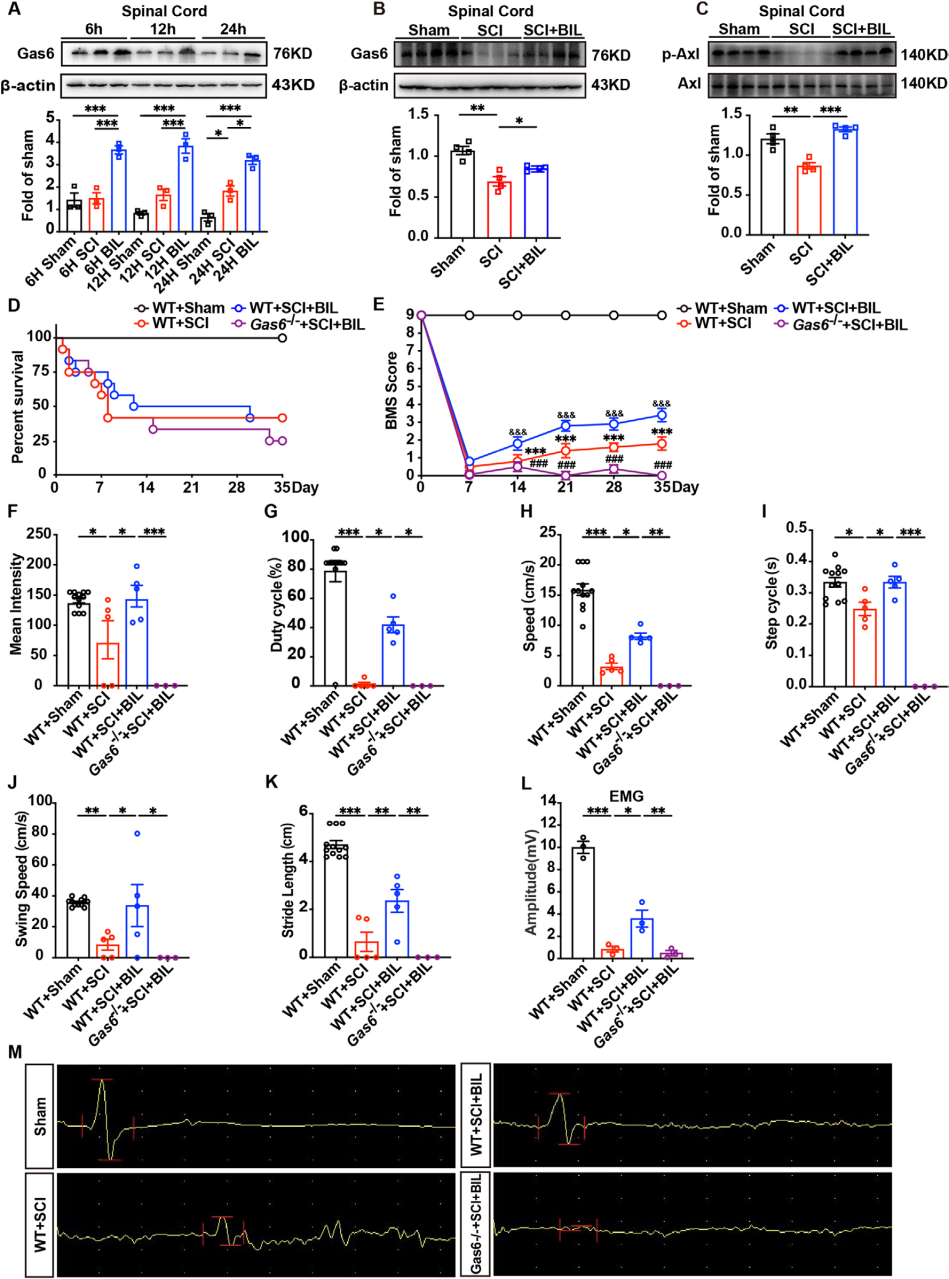
(A) Gas6 protein levels in the spinal cord were measured in sham, SCI, and SCI + BIL groups at 6, 12, and 24 h post‐SCI using western blot (*n* = 3). (B‐C) Gas6 and p‐Axl protein levels in the spinal cord were measured in sham, SCI, and SCI + BIL groups at 35 days after SCI using western blot (*n* = 4). (D) The survival rate of mice in the Sham group, WT + SCI, WT + SCI + BIL, and Gas6‐/‐ + SCI + BIL groups. Survival was assessed over a period of 35 days post‐SCI. (E) Basso Mouse Scale (BMS) scores were used to assess locomotor function over 5 weeks post‐SCI. (F‐K) Quantitative analysis of gait parameters using the CatWalk system in each group. The following gait parameters were quantitatively analyzed: (F) mean intensity, (G) Duty cycle, (H) speed, (I) step cycle, (J) swing speed, and (K) stride length. (L) Quantification analysis of amplitude of EMG in each group (*n* = 3). (M) Representative EMG images were obtained 35 days post‐SCI (*n* = 3). Data are presented as mean ± SEM. *p < 0.05, **p < 0.01, ***p < 0.001.

As shown in Figure 4A, mice were treated with bilirubin at 6, 12, and 24 h after SCI surgery. Western blot analysis revealed that bilirubin significantly increased the level of Gas6 in the spinal cord within a short period of time. Further investigation demonstrated that bilirubin not only elevated Gas6 levels but also induced the activation of Axl in the spinal cord of mice at 35 days post‐SCI (Figure 4B,C).

More importantly, we also found that knockout of the Gas6 gene can cancel the therapeutic effect of bilirubin in mice. For example, the *Gas6* ^*−*^ */* ^*−*^ + SCI + BIL group exhibited a significantly higher mortality rate and lower BMS score compared to the WT + SCI + BIL group (Figure 4D,E). Gait analysis conducted using the CatWalk system corroborated these findings. The WT + SCI + BIL group mice exhibited notable enhancements in gait parameters, including mean intensity, footprint area, duty cycle, speed, stand time, step cycle, swing speed, and stride length. However, in the *Gas6* ^*−*^ */* ^*−*^ + SCI + BIL group, bilirubin failed to produce comparable improvements in these gait parameters, suggesting that Gas6 is essential for bilirubin's beneficial impact on gait and motor coordination in SCI (Figure 4F–M). Additionally, EMG analysis demonstrated that bilirubin treatment significantly increased EMG amplitude; however, this effect was markedly diminished in the *Gas6* ^*−*^ */* ^*−*^ + SCI + BIL group (Figure 4N,O). Collectively, these results highlight the critical role of Gas6 in mediating the neuroprotective and functional recovery effects of bilirubin for SCI, suggesting that Gas6 is an indispensable component of bilirubin's anti‐inflammatory and motor recovery mechanisms.

**FIGURE 5:**
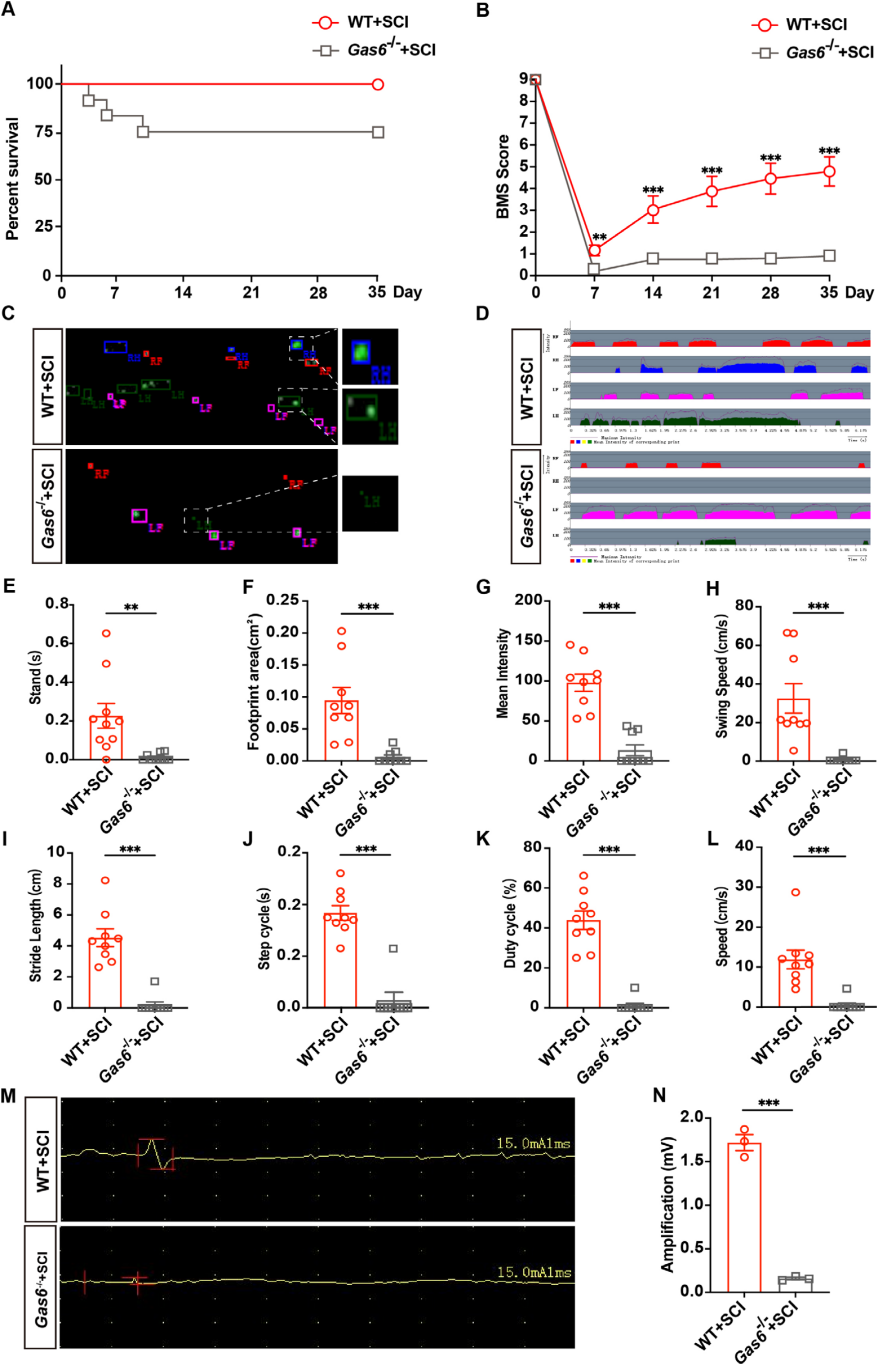
(A) The survival rate of mice in the WT + SCI and Gas6‐/‐ + SCI groups. Survival was assessed over a period of 35 days post‐SCI (*n* = 9). (B) Basso Mouse Scale (BMS) scores used to assess locomotor function over 5 weeks post‐SCI (*n* = 9). (C) Representative footprint intensity images for each group were obtained at 35 days post‐SCI (*n* = 9). (D) Footprint mean intensity images for each group were captured at 35 days post‐SCI (*n* = 9). (E‐L) Quantitative analysis of gait parameters using the CatWalk system in each group. Significant reductions in gait analysis parameters were observed in the Gas6‐/‐ + SCI group compared to the WT + SCI group at 35 days post‐SCI. The following gait parameters were quantitatively analyzed: (E) stand duration, (F) footprint area, (G) mean intensity, (H) swing speed, (I) stride length, (J) step cycle, (K) duty cycle and (L) speed (*n* = 9). (M) EMG images for each group were recorded at 35 days post‐SCI (*n* = 3). (N) Quantitative analysis of EMG amplitude was performed for each group (*n* = 3). Data are presented as mean ± SEM. *p < 0.05, **p < 0.01, ***p < 0.001.

To further confirm the importance of Gas6 in SCI, we conducted the following experiment. We reduced the intensity of the injury from 60 kilodynes to 40 kilodynes. The results showed that, despite the reduced injury intensity, the *Gas6* ^*−*^ */* ^*−*^ mice did not show any significant recovery. In contrast, WT mice exhibited significant recovery under this lower intensity injury. Firstly, survival analysis revealed that *Gas6* ^*−*^ */* ^*−*^ mice exhibited a significantly higher mortality rate compared to WT mice following SCI. The WT group showed 100% survival; the *Gas6* ^*−*^ */* ^*−*^ group experienced a notable decline with 75% survival during the 5‐week observation period (Figure 5A). Furthermore, *Gas6* ^*−*^ */* ^*−*^ mice exhibited a lower BMS score (Figure 5B). Representative footprints and gait patterns showed that *Gas6* ^*−*^ */* ^*−*^ mice had abnormal gait patterns with reduced footprint area and irregular walking patterns compared to WT mice (Figure 5C,D). These findings were further supported by quantification of various gait parameters. Gas6^*−*^ */* ^*−*^ mice exhibited a significantly shorter stand time (Figure 5E), reduced footprint area (Figure 5F), and lower mean intensity (Figure 5G) compared to WT mice. Moreover, swing speed (Figure 5H), stride length (Figure 5I), step cycle (Figure 5J), duty cycle (Figure 5K) and overall speed (Figure 5L) were markedly impaired in *Gas6* ^*−*^ */* ^*−*^ mice. Additionally, EMG analysis demonstrated that *Gas6* ^*−*^ */* ^*−*^ mice significantly decreased EMG amplitude (Figure 5M,N).

## 4 Discussion

This study demonstrates that bilirubin, a metabolite long regarded as a waste product of heme catabolism, exhibits significant neuroprotective and anti‐inflammatory properties in the context of SCI. Our findings reveal that bilirubin administration improves motor function in SCI mice, an effect closely linked to the upregulation of SOCS3 expression. Furthermore, bilirubin alleviates neuroinflammation through activation of the Gas6/Axl–SOCS3 signaling pathway, providing novel mechanistic insights into its therapeutic potential. The TAM receptor family (Tyro3, Axl, and Mer), particularly the Gas6/Axl axis, plays crucial roles in neuroprotection across various neurological conditions. In Alzheimer's disease models, this pathway has been shown to attenuate Aβ‐induced neurotoxicity and reduce tau hyperphosphorylation, whereas in our SCI model it mediates bilirubin's anti‐inflammatory effects through SOCS3 induction, a critical suppressor of pro‐inflammatory pathways. These results align with growing recognition of bilirubin's role as an endogenous antioxidant and immunomodulator in neurological disorders, where it has been shown to mitigate oxidative stress and inflammatory responses in conditions ranging from stroke to neurodegenerative diseases [31, 32].

The anti‐inflammatory effects of bilirubin appear to be mediated through the Gas6/Axl‐SOCS3 signaling axis. The TAM receptor family (Tyro3, Axl, and Mer) plays a crucial role in efferocytosis and the regulation of immune responses, helping to maintain tissue homeostasis by preventing excessive inflammation [33, 34, 35]. SOCS3, a downstream mediator of TAM receptor signaling, is critical for suppressing pro‐inflammatory pathways [12, 28]. Our study shows that bilirubin markedly upregulates Gas6 and Axl expression, leading to SOCS3 induction. Notably, Gas6 deficiency or Axl inhibition abolished bilirubin's therapeutic effects, confirming that its neuroprotective action depends on the Gas6/Axl/SOCS3 pathway. Although our focus was on microglial Gas6/Axl/SOCS3 signaling, we acknowledge that systemic Gas6 knockout may affect other cell types (e.g., endothelial cells, macrophages) involved in SCI pathophysiology. Thus, the observed phenotype could reflect contributions from multiple cell lineages. Future studies using cell‐specific knockout models will help dissect these roles.

Despite these promising findings, translating bilirubin‐based therapies for SCI faces considerable challenges. The compound's well‐established neurotoxicity at elevated concentrations, as seen in kernicterus, necessitates careful optimization of dosing regimens and delivery methods to maximize therapeutic benefits while minimizing systemic exposure. Future research should prioritize developing targeted spinal cord delivery systems to avoid potential off‐target effects in other organs such as the liver and brain. Additionally, whereas our study establishes the functional significance of Gas6 in acute SCI recovery, several important questions remain unanswered. The 5‐week observation period, whereas sufficient to capture key acute and subacute processes, precludes definitive conclusions about chronic pathology. Longer‐term studies extending beyond 12 weeks will be essential to evaluate glial scar maturation and sustained functional outcomes. Furthermore, although we identified the Gas6/Axl/SOCS3 pathway in microglial cell lines, in vivo validation using techniques such as FACS‐sorted spinal cord microglia or microglia‐specific immunofluorescence will be critical for confirming cell‐type‐specific signaling mechanisms. Perhaps most importantly, the precise molecular interaction between bilirubin and the Gas6/Axl system remains to be fully elucidated. Complementary approaches including co‐immunoprecipitation, surface plasmon resonance, and structural biology studies will be required to determine whether bilirubin directly binds Gas6/Axl or modulates this pathway through indirect mechanisms.

**FIGURE 6:**
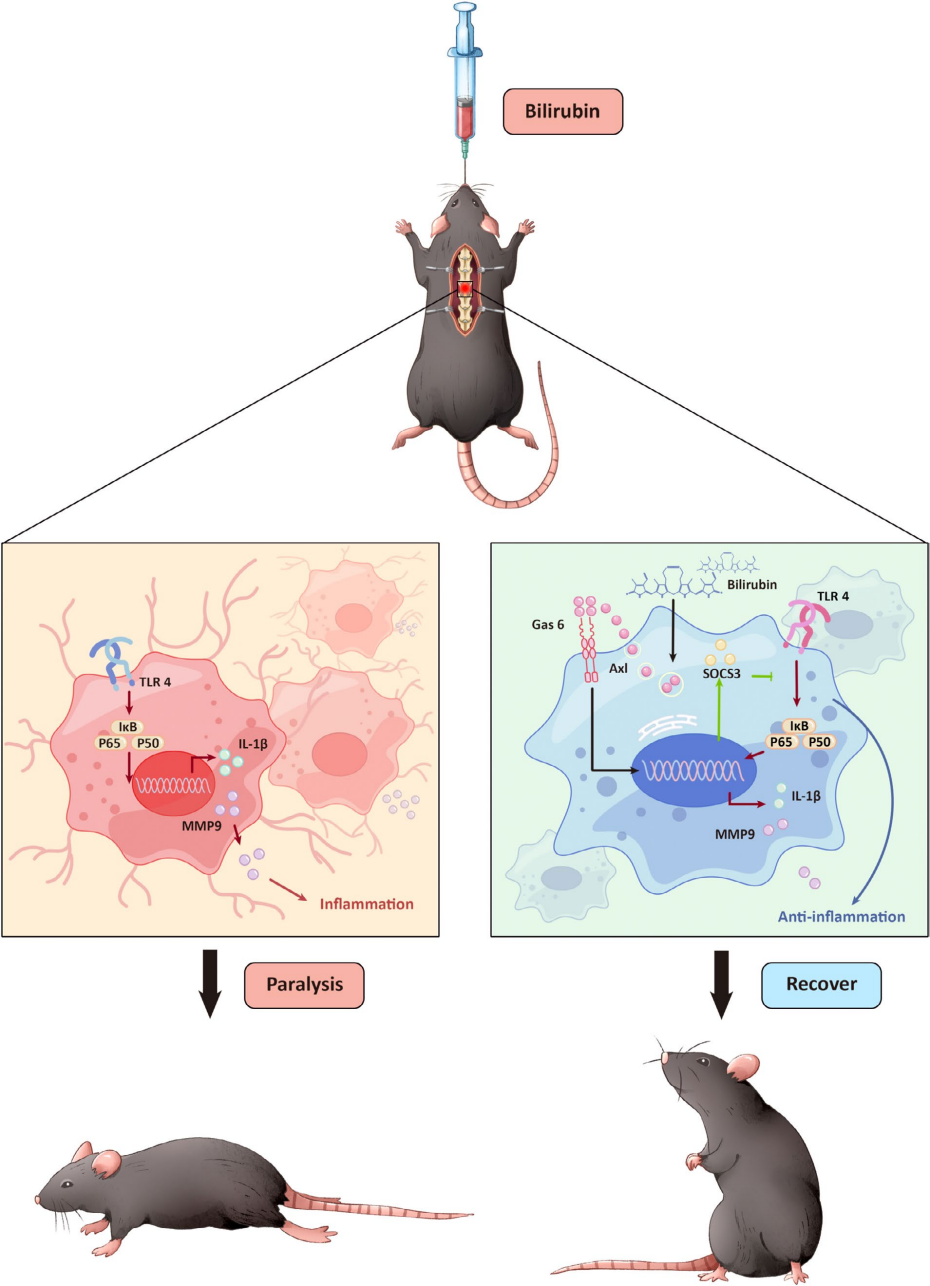
Schematic illustration showing that bilirubin activates Gas6/Axl/SOCS3 signaling to alleviate neuroinflammation and promote recovery from SCI.

In conclusion, our work provides the first evidence that bilirubin exerts neuroprotective effects in SCI through activation of the Gas6/Axl‐SOCS3 signaling axis, resulting in suppression of neuroinflammation and improvement of functional recovery (Figure 6). These findings not only expand our understanding of bilirubin's physiological roles beyond its traditional classification as a metabolic byproduct, but also identify a novel therapeutic target for modulating neuroinflammatory responses in SCI. Although significant translational hurdles remain, this study lays important groundwork for future investigations aimed at harnessing bilirubin's therapeutic potential while overcoming its inherent limitations for clinical application.

## Author Contributions

R.W., T.‐T.L., and D.‐Y.P. designed and supervised the research. K.‐M.J. and Y.‐Q.L. did laboratory experiments and drafted the manuscript. N.S., Y.W., and X.‐T.Q. performed data analysis. K.‐M.J., W.‐T.L., and L.H. verified the data. All authors discussed the data and contributed to the final version of the manuscript.

## Consent

The authors have nothing to report.

## Conflicts of Interest

The authors declare no conflicts of interest.

## Supporting information


**Data S1:** cns70538‐sup‐0001‐DataS1.pdf.

## Data Availability

All data supporting the findings of this study are available within the article from the corresponding author upon reasonable request.
