# Supplementary material for: Bilirubin Alleviates Spinal Cord Injury by Enhancing SOCS3‐Mediated Anti‐Inflammatory Effects via Gas6‐Axl Signaling
Source: CNS Neurosci Ther. 2025 Sep 17;31(9):e70538. doi: 10.1111/cns.70538 (PMC12441303; doi:10.1111/cns.70538)

Full unedited blot for Figure2C

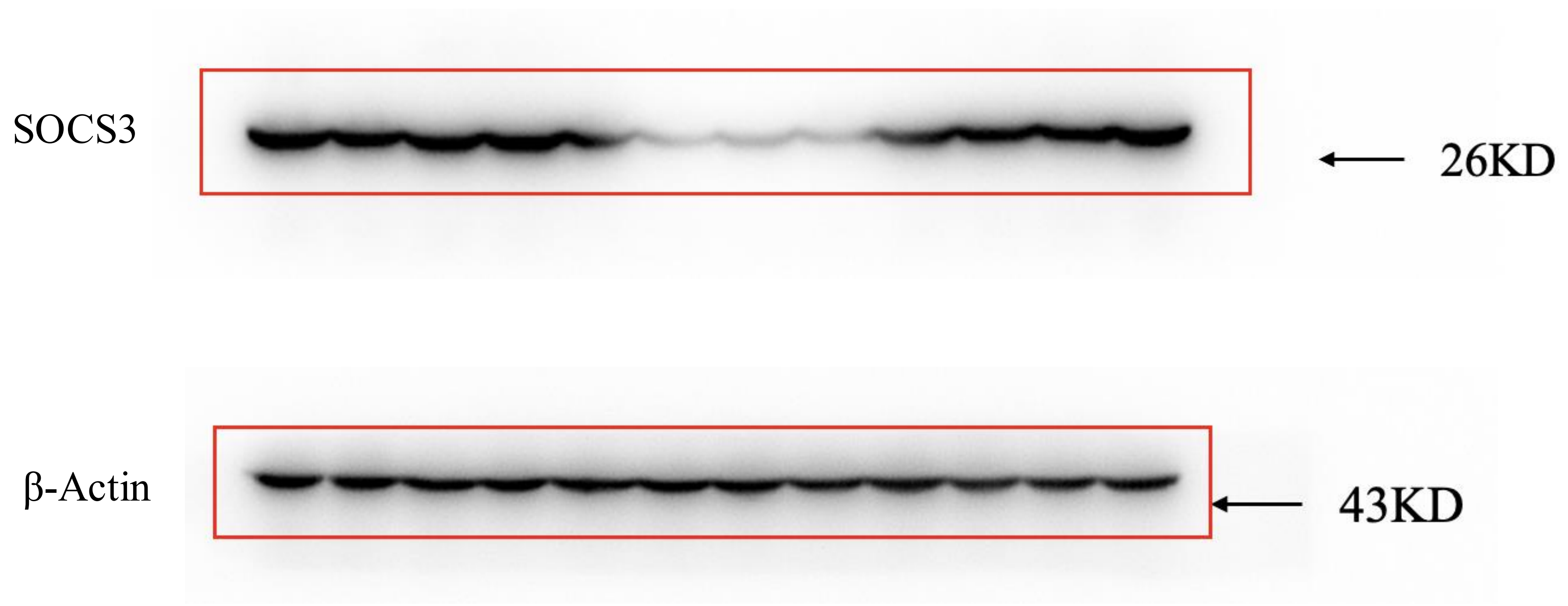

Full unedited blot for Figure2D

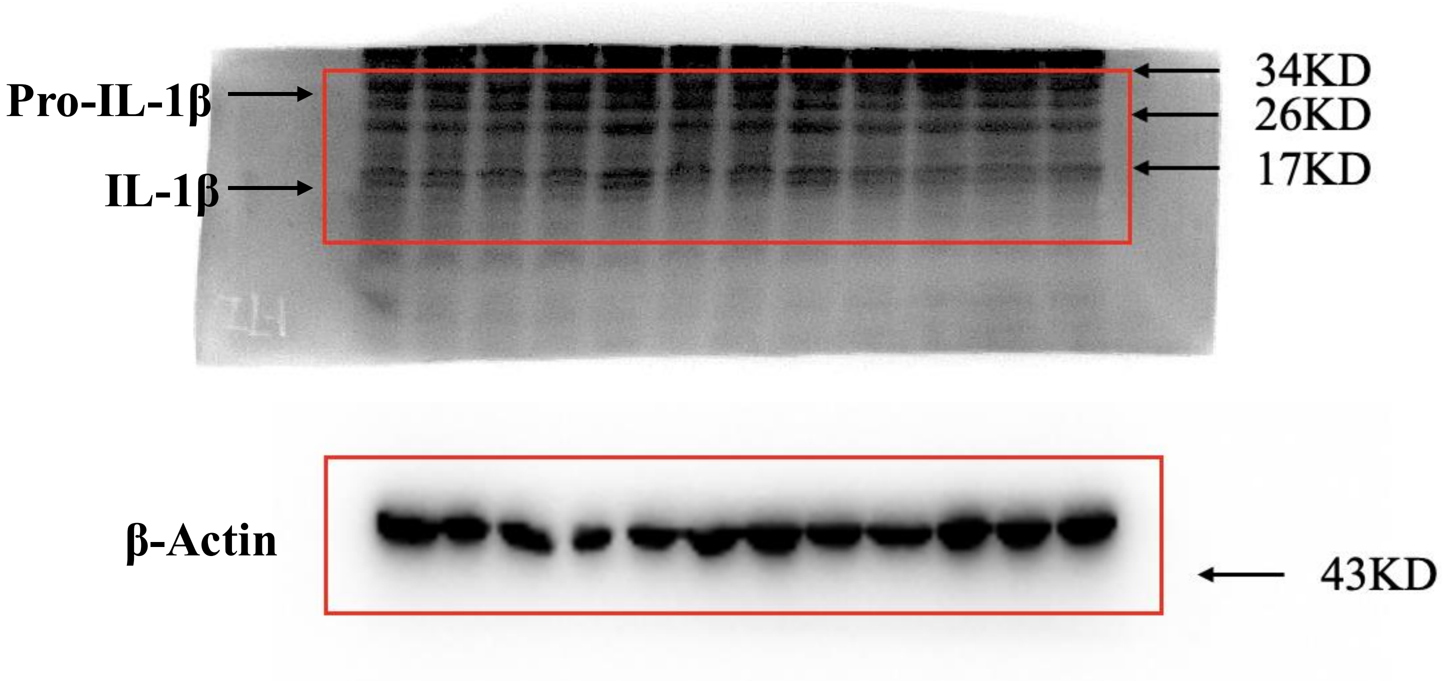

Full unedited blot for Figure2E

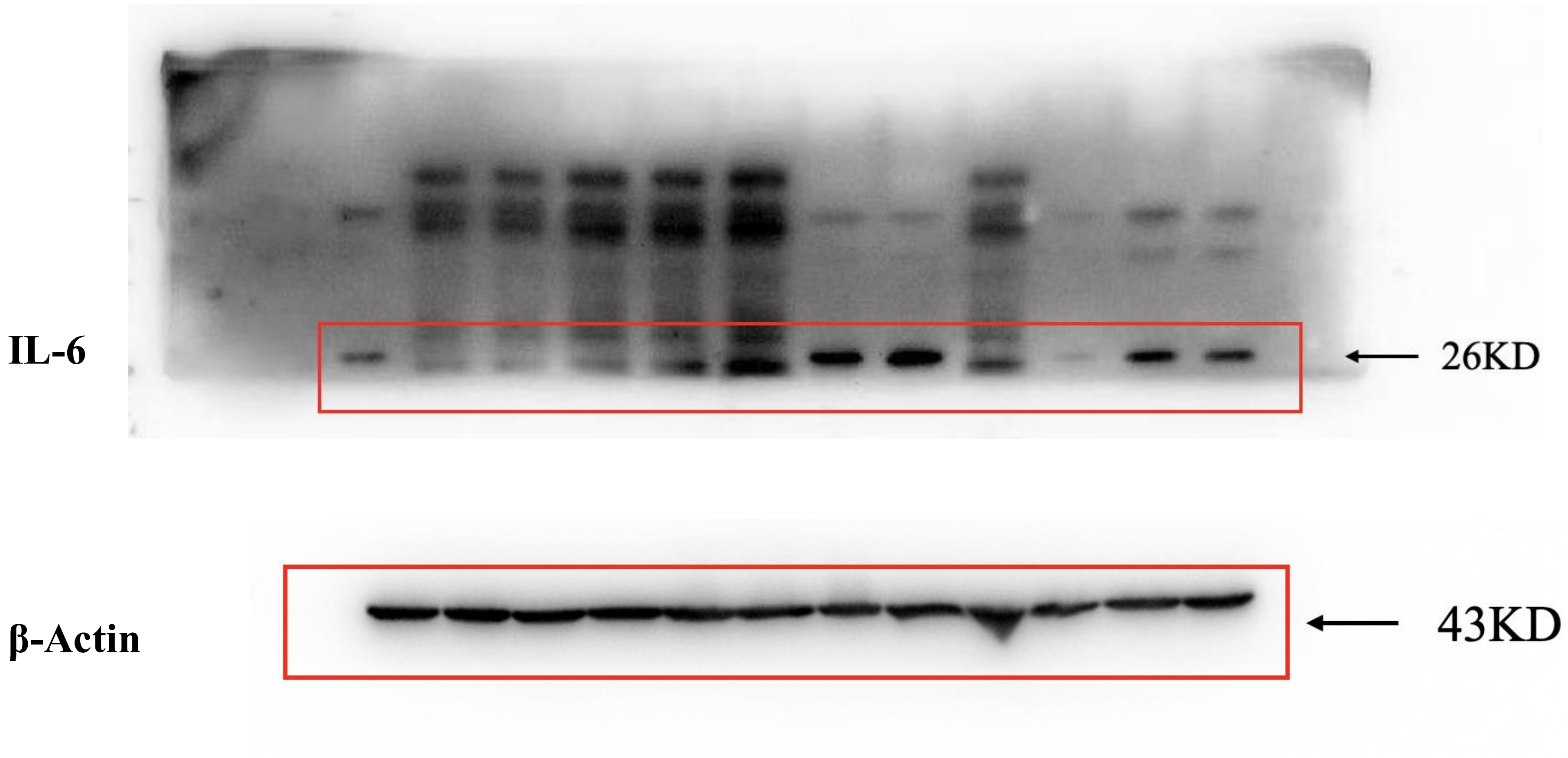

Full unedited blot for Figure2F

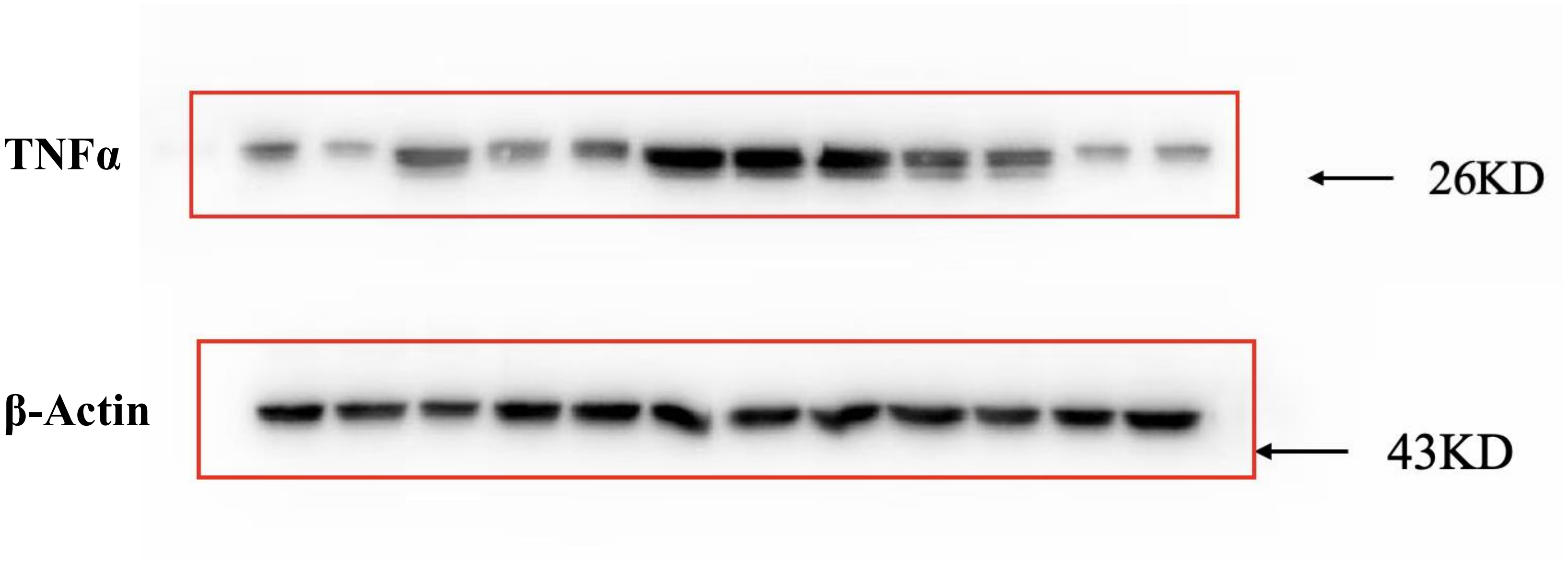

Full unedited blot for Figure2G

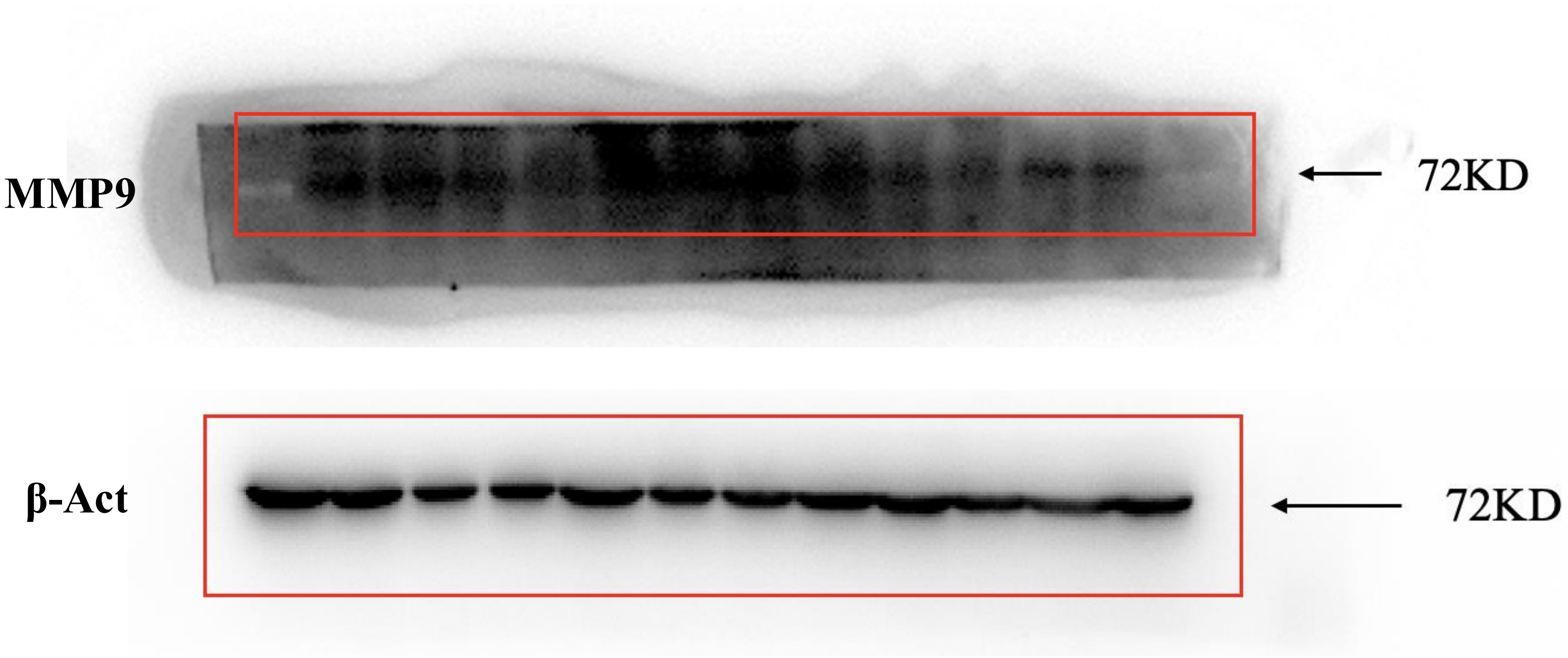

Full unedited blot for Figure3A

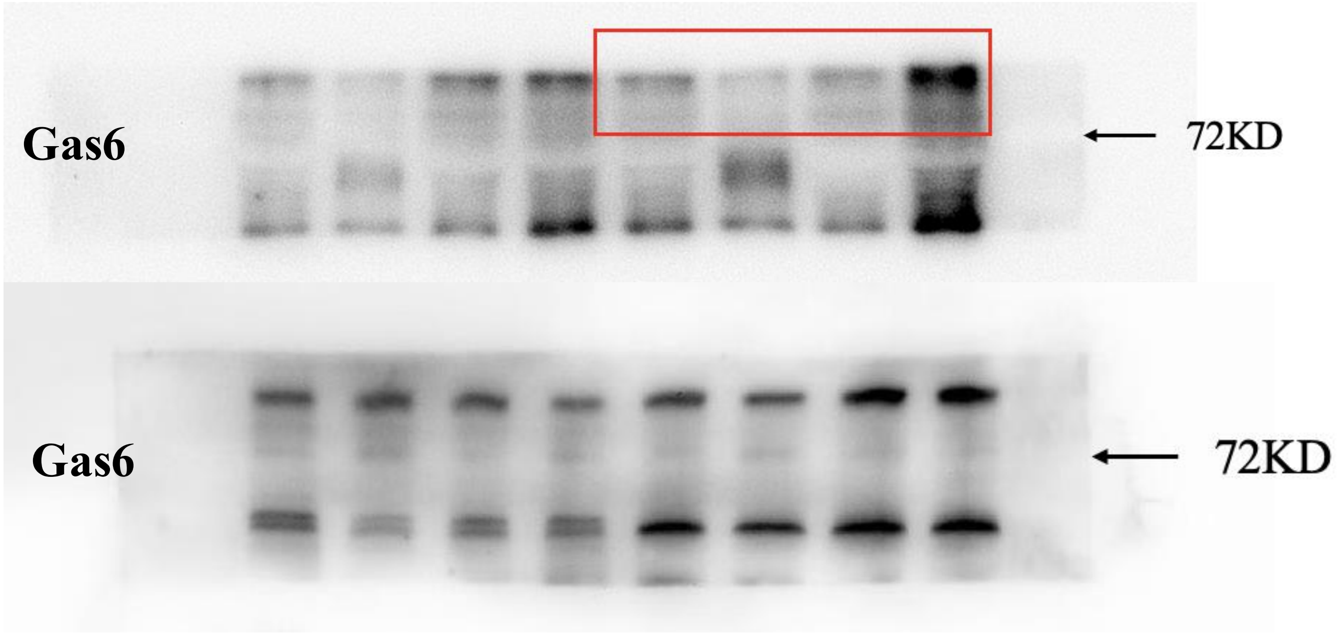

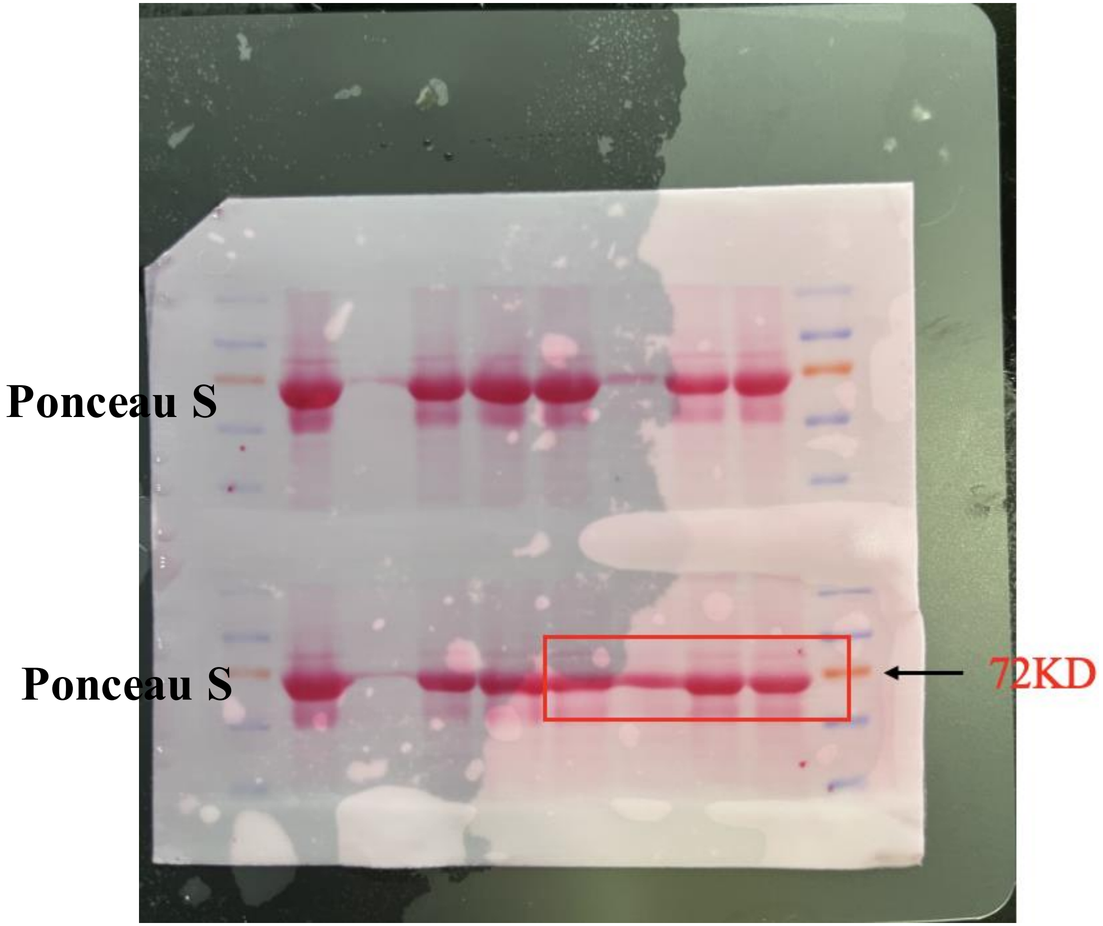

Full unedited blot for Figure3B

Axl

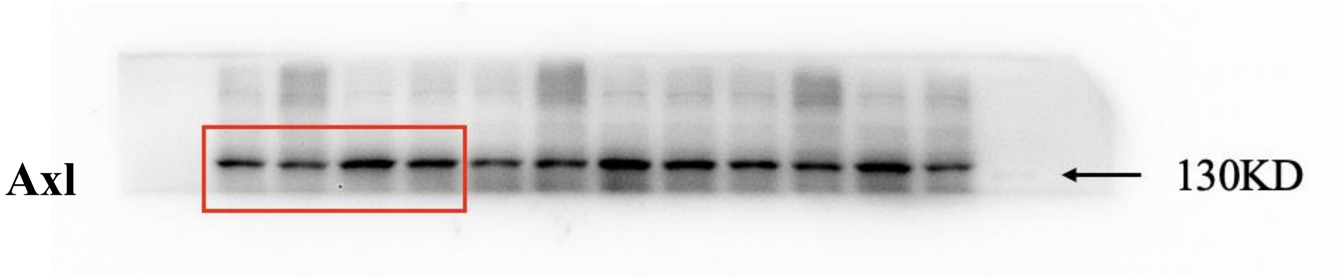

← 130KD

Axl

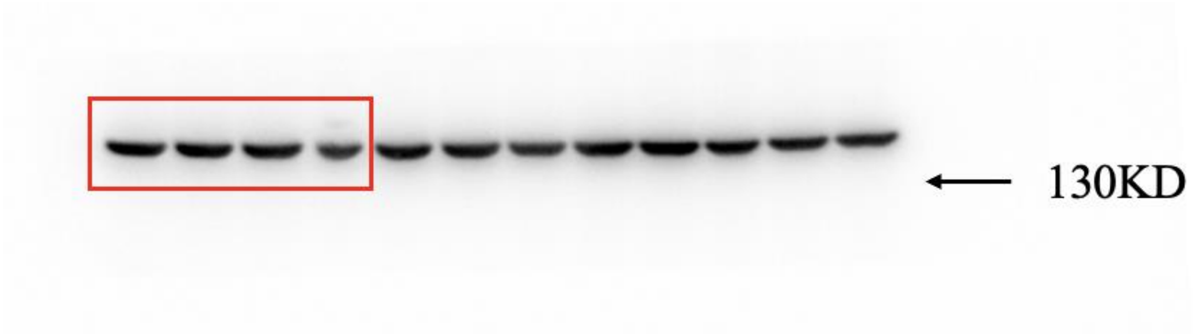

← 130KD

Full unedited blot for Figure3C: SOCS3

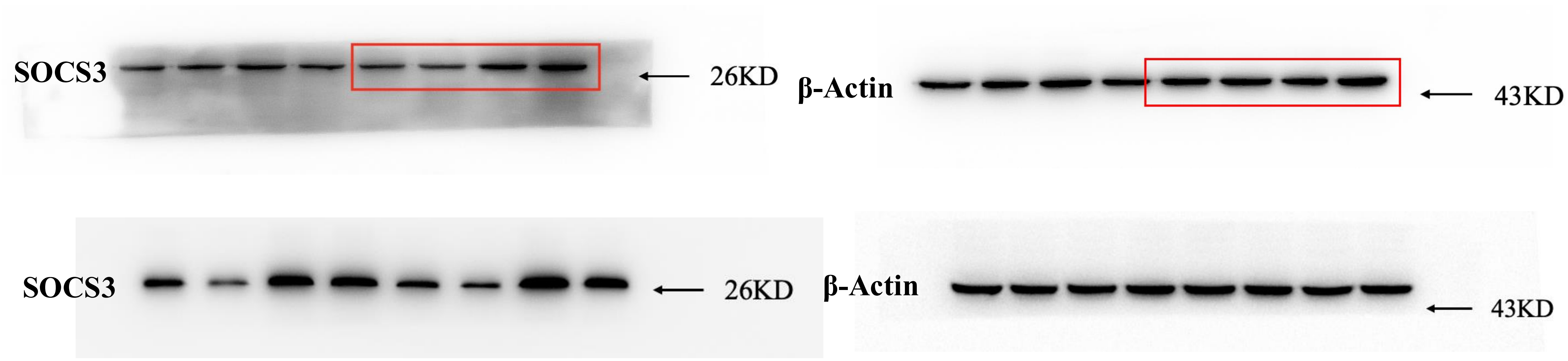

Full unedited blot for Figure3D

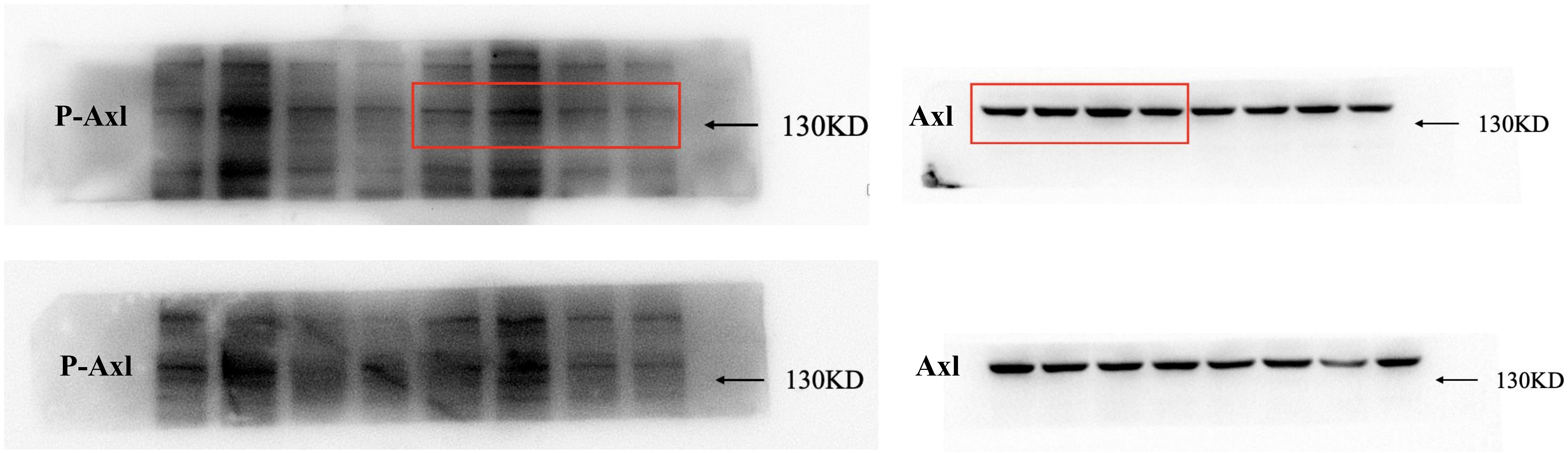

Full unedited blot for Figure3E

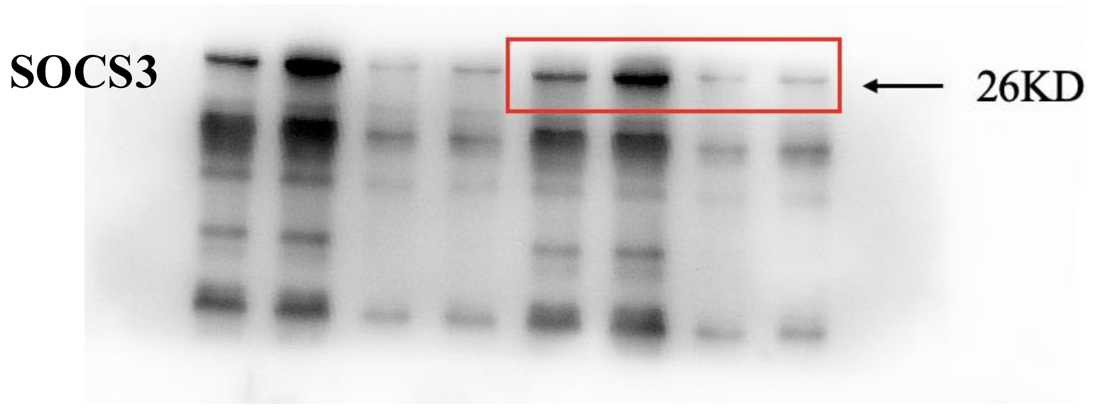

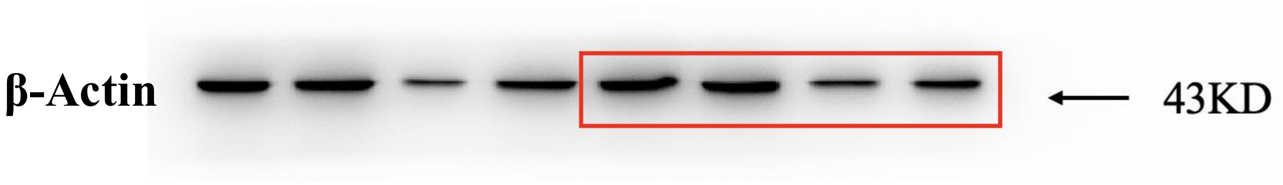

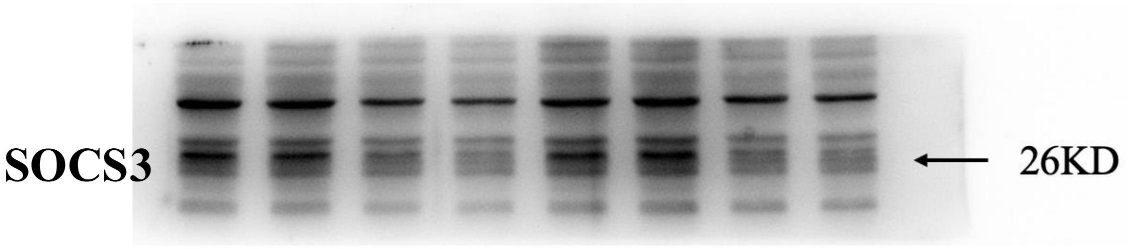

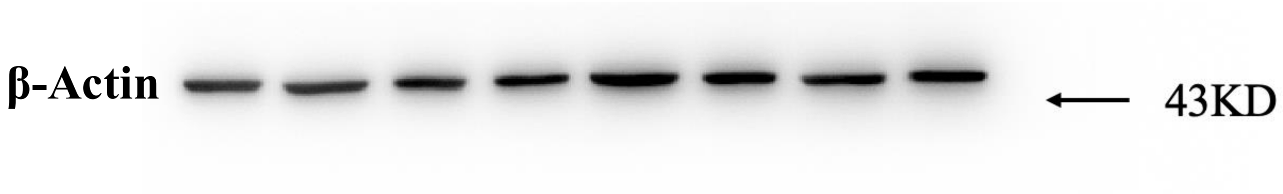

Full unedited blot for Figure3F

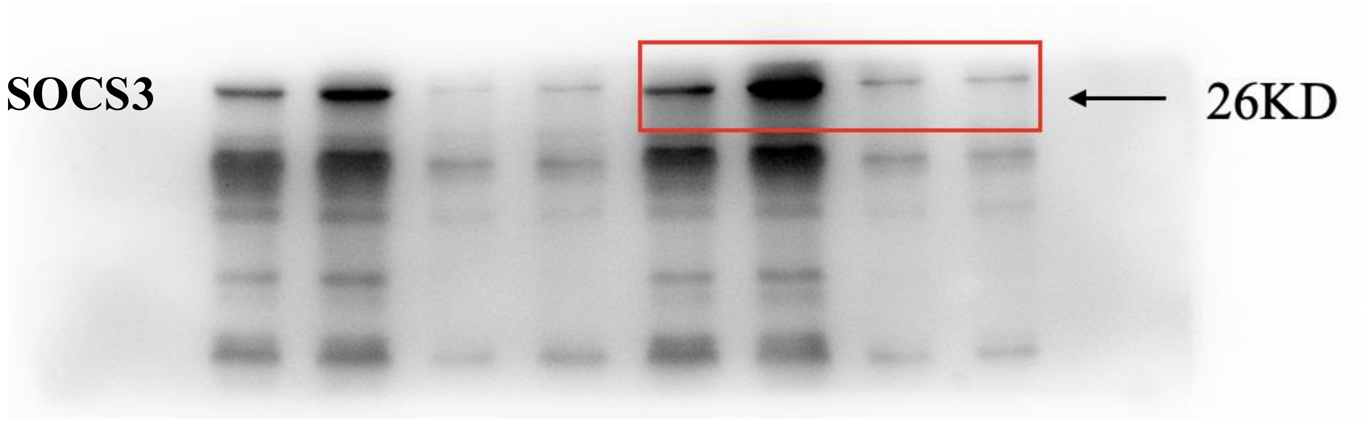

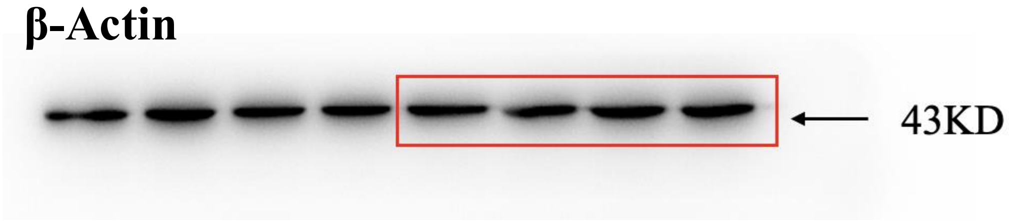

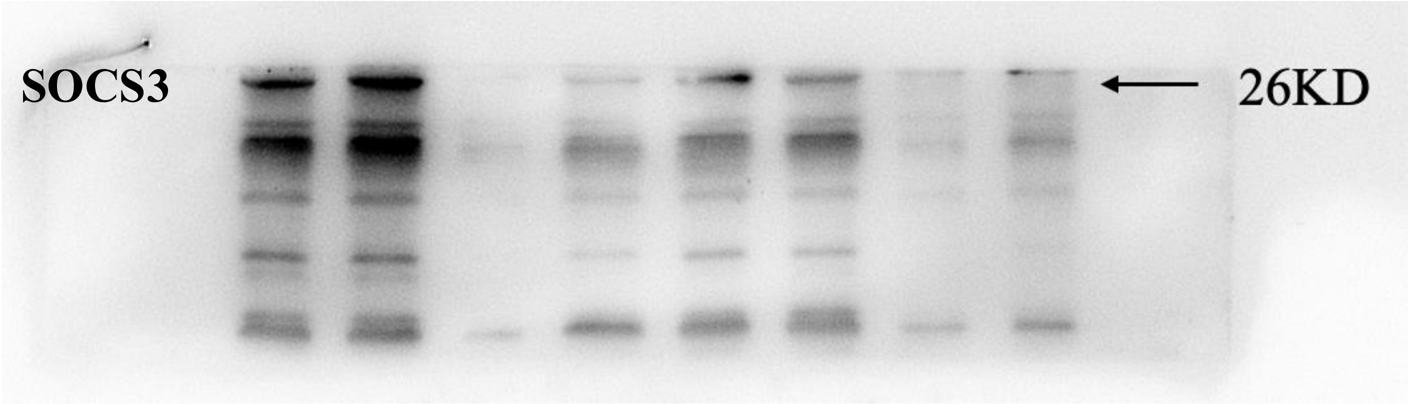

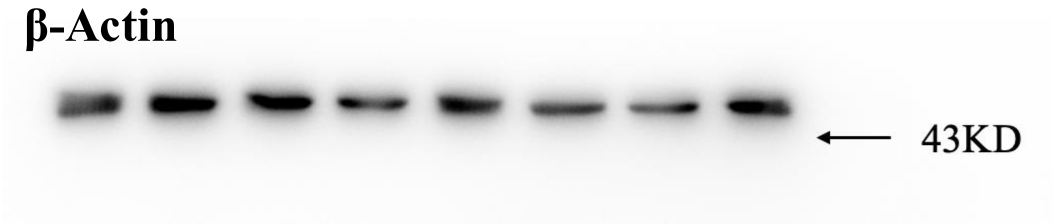

Full unedited blot for Figure4A

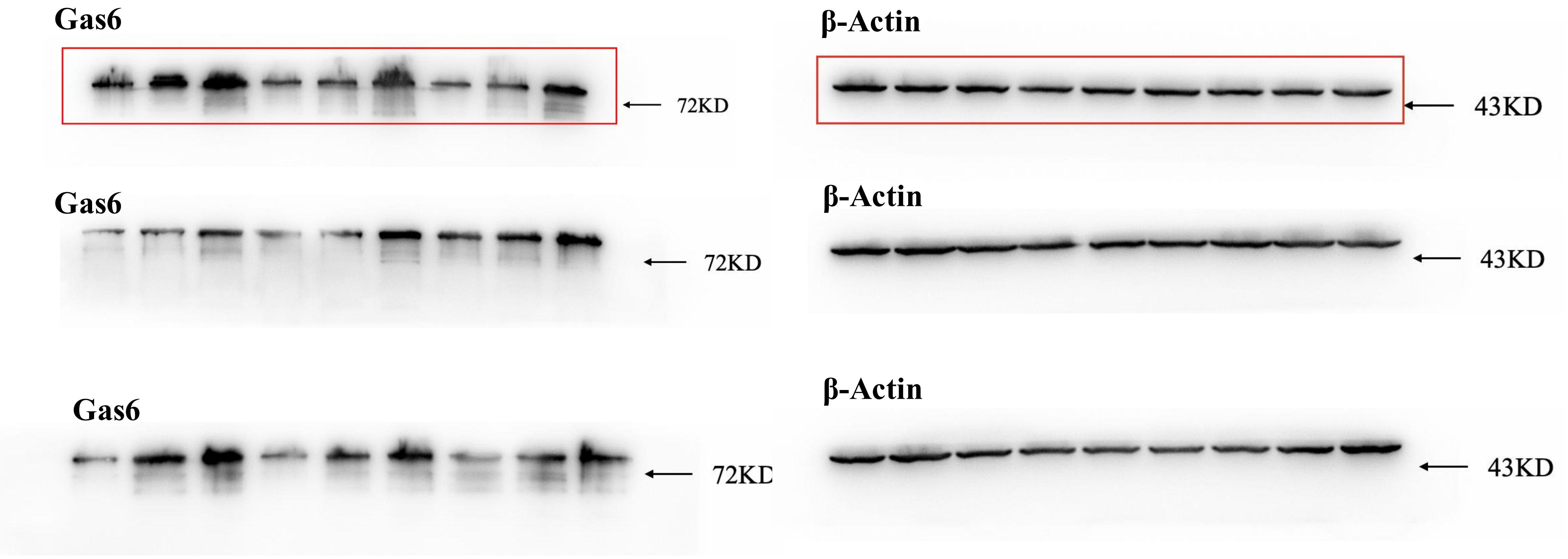

Full unedited blot for Figure4B: Gas6

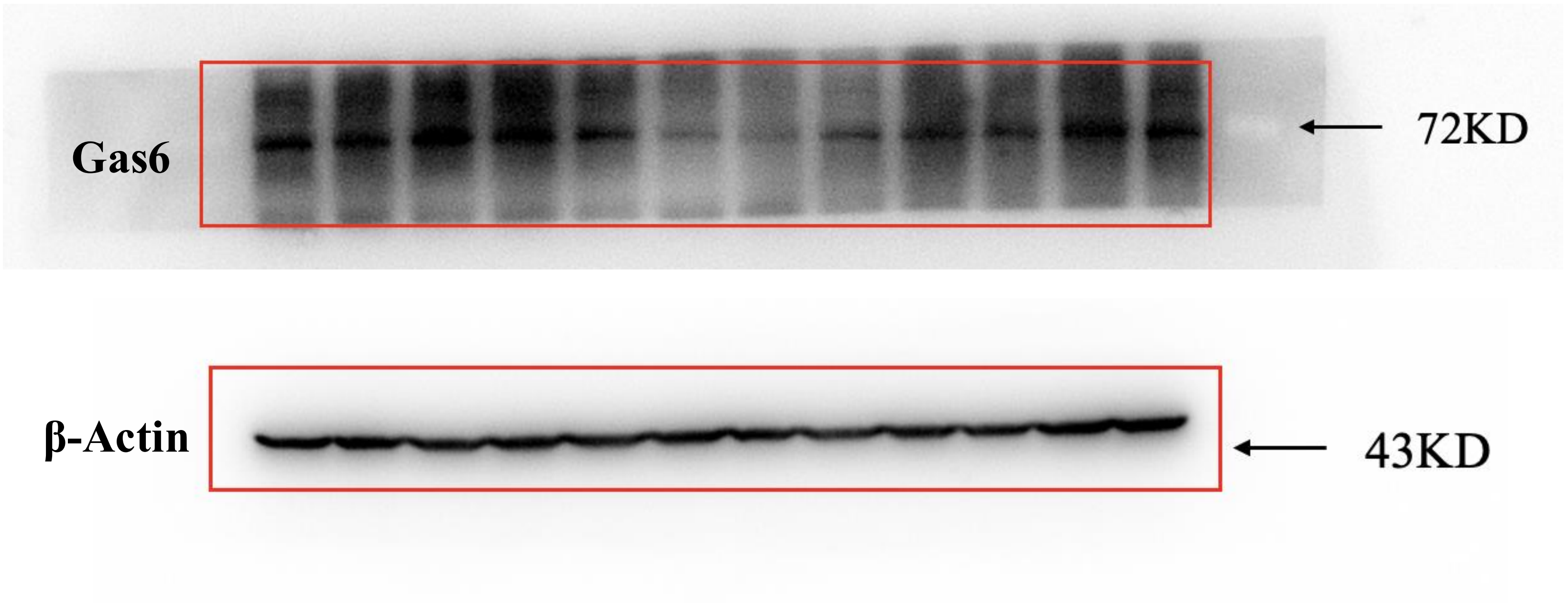

Full unedited blot for Figure4C: p-Axl

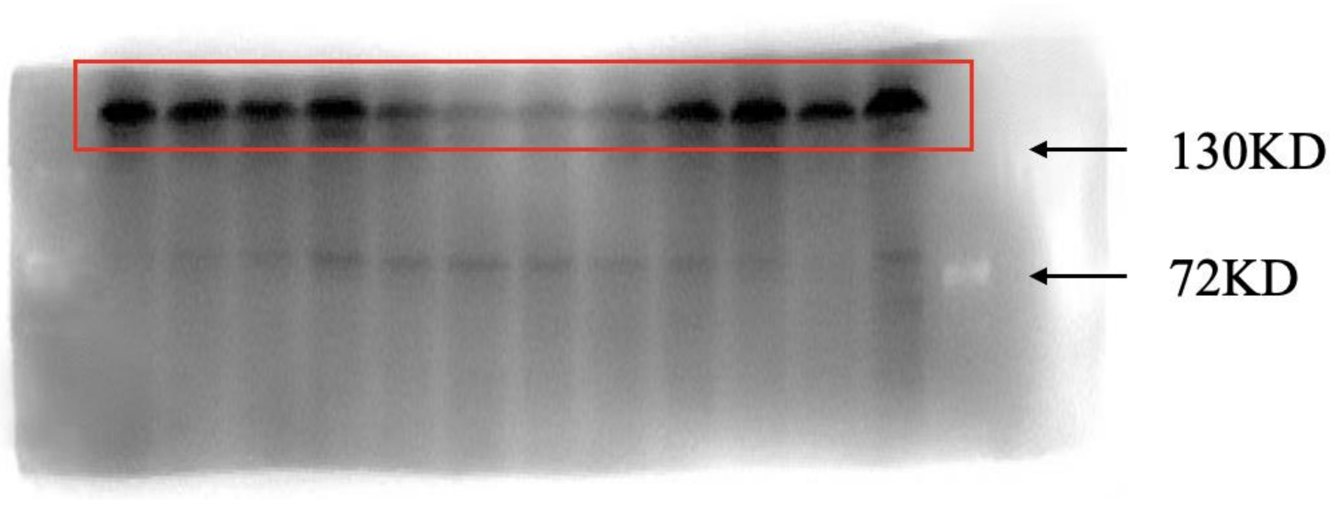

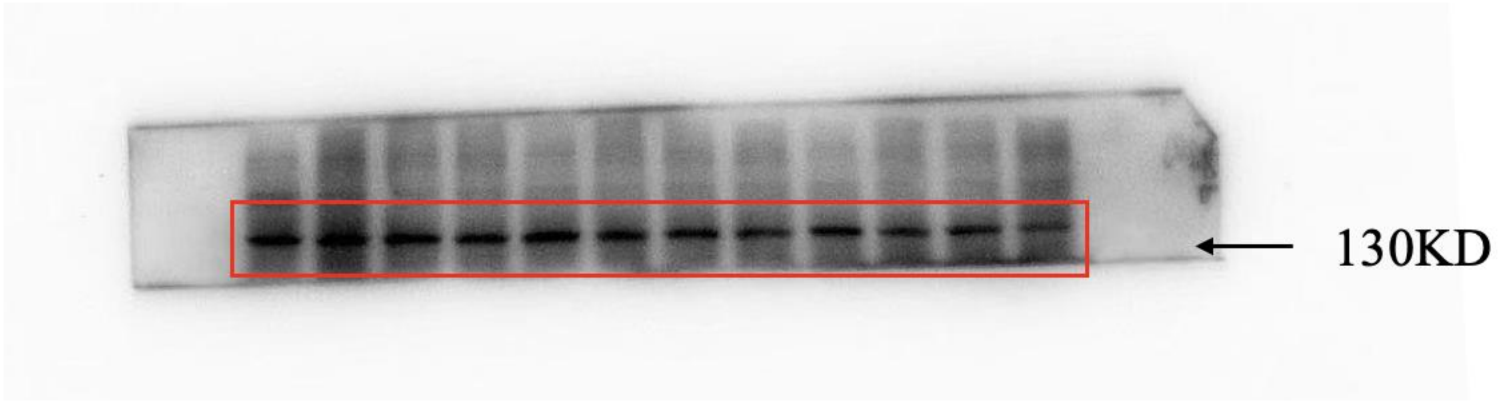

Supplement: Supplementary file 1 — Data S1: cns70538‐sup‐0001‐DataS1.pdf. [file CNS-31-e70538-s001.pdf]
